# Utility estimation of hypothetical chronic obstructive pulmonary disease health states by the general population and health professionals

**DOI:** 10.1186/s12955-015-0228-2

**Published:** 2015-03-13

**Authors:** Sujin Cho, Hochang Kim, Seon-Ha Kim, Minsu Ock, Yeon-Mok Oh, Min-Woo Jo

**Affiliations:** Asan Medical Center, Seoul, South Korea; University of Ulsan College of Medicine, Seoul, South Korea; Department of Nursing, College of Health Science, Dankook University, Cheonan, South Korea; Department of Preventive Medicine, University of Ulsan College of Medicine, 88, Olympicro-43gil, Songpa-gu, Seoul, 138-736 South Korea; Department of Pulmonary and Critical Care Medicine and Clinical Research Center for Chronic Obstructive Airway Diseases, Asan Medical Center, University of Ulsan College of Medicine, Seoul, Korea

**Keywords:** Chronic obstructive pulmonary disease, Quality of life, Utility

## Abstract

**Purpose:**

This study attempted to estimate the utility weights for hypothetical chronic obstructive pulmonary disease (COPD) health states, including the effect of exacerbation, and based on utilities elicited from a representative sample using the time trade-off (TTO).

**Methods:**

A total of 200 study subjects were recruited using a quota sampling method in Seoul, Korea. Ten COPD health profiles were described reflecting the severity of COPD and the extent of exacerbation. Respondents evaluated each health state using a visual analogue scale and TTO during a personal interview. TTO values were estimated using a linear mixed model, and the model performance was evaluated in terms of its predictive ability and goodness of fit.

**Results:**

The estimated TTO values were 0.824 in moderate, 0.646 in severe, and 0.305 in very severe COPD health states. The estimated utility decrements in TTO varied from 0.082 for a non-serious exacerbation to 0.228 for one non-serious plus one serious exacerbation per year. The mean absolute error of the TTO model was 0.008, and the generalized R^2^ was 0.86.

**Conclusion:**

The social preference of various COPD health states and the utility decrement due to exacerbation can be useful for the economic evaluation of COPD intervention in Korea.

**Electronic supplementary material:**

The online version of this article (doi:10.1186/s12955-015-0228-2) contains supplementary material, which is available to authorized users.

## Introduction

Chronic obstructive pulmonary disease (COPD) is characterized by persistent airflow limitation which is usually progressive and associated with an enhanced chronic inflammatory response in the airway and lung to noxious particles or gases. COPD is a leading cause of morbidity and mortality worldwide, and its prevalence and mortality rates are currently rising [1]. As a result, the increasing economic and social burden of COPD is substantial [2]. COPD patients have a low health-related quality of life (HRQOL), especially in terms of physical, emotional, and mental health [3].

HRQOL refers to the physical, psychological, and social domains of health which are seen as distinct areas that are influenced by a person’s experiences, beliefs, expectations, and perceptions [4]. Utility is a preference that individuals or societies may have for any particular set of health outcomes and which is useful in measuring HRQOL adjustment to a given set of treatment outcomes [5]. This utility is used for economic evaluation such as cost-utility analysis (CUA), medical decision making in a clinical setting, and evaluation and monitoring of population health [6]. Utilities are obtained either via direct valuation techniques, such as standard gamble, time trade-off (TTO), and the use of a rating scale, or indirectly via generic preference-based life instruments such as EQ-5D.

COPD is characterized by fluctuations in symptoms and quality of life, although the disease generally progresses slowly over a period of many years [7]. The goal of COPD management includes symptom relief and risk, i.e. disease progression, exacerbation, and mortality, reduction [2]. Utility measurements of COPD health states, according to the disease severity and exacerbation, are required in order to conduct economic evaluation regarding COPD intervention. In previous studies, utilities in different COPD states and the impact of disease exacerbation varied substantially [8-10]. Evidence suggests that the EQ-5D for COPD and its exacerbations has limited discriminatory ability, particularly between moderate and severe COPD, and that the responsiveness to clinically relevant changes in stable COPD over time, due to treatment, also appears limited [11-13]. This utility may also differ according to the perspective or type of interviewee, such as patients, physicians or the general population [14].

The purposes of this study are to elicit a social value for COPD health states that describe the commonly occurring combination of COPD severity and exacerbation patterns through TTO and the visual analogue scale (VAS) and to develop a prediction model to estimate the utility weights of COPD states in the Korean population. We also attempted to compare the utility weights between the general population and health professionals.

## Methods

### Study population

This study was approved by the Institutional Review Board of Asan Medical Center (approval number: 2011–0119). The target general population consisted of adults aged 19 years or older living in Seoul, Korea. Quota sampling was used and proportionally represented the 2010 national census in terms of age and sex. All participants provided written informed consent. Each respondent was asked to complete the questionnaire during a face-to-face interview. The survey was performed from 13 April 2012 to 11 May 2012 by 10 interviewers trained in evaluating methods. Health professionals involved in the care of COPD patients were also interviewed regarding the same evaluation task.

### Questionnaire and health profiles

The questionnaire consisted of questions regarding demographic factors, i.e. sex, age, and level of education, and the self-perceived health status using EQ-5D, followed by evaluation of 10, COPD health profiles using both the VAS and TTO methods. The 10, COPD health profiles were developed based on the study by Rutten-van Mölkenet al. [7] and were modified after consultation with a respiratory specialist. Each health profile included one of the three levels of severity of COPD, i.e. mild, moderate or severe COPD approximately corresponding to a grade of II, III or IV, respectively, based on the Global Initiative for Chronic Obstructive Lung Disease [GOLD] grades [15] as well as information regarding the frequency and severity of symptom exacerbation. The COPD severity was described in terms of six dimensions: (i) extent of dyspnea; (ii) impact on non-strenuous activities; (iii) impact on strenuous activities; (iv) ability to work; (v) anxiety and depression; and (vi) energy and tiredness. Each dimension consisted of three levels (Additional file 1: Appendix 1). The exacerbation extent was categorized as non-serious and serious exacerbation. Exacerbation was operationally described in terms of three aspects: (i) symptoms in both the respiratory and non-respiratory system and impact on daily activities; (ii) required treatment; and (iii) symptom duration (Additional file 1: Appendix 2).

In this study, each COPD health status was expressed as a three-digit number, with the first digit indicating whether the patient suffered from moderate (1), severe (2) or very severe (3) COPD. The second digit indicates the number of non-serious exacerbations (0 or 1), and the third digit indicates the number of serious exacerbations (0 or 1). For example, the profile 211 refers to moderate COPD with one non-serious and one serious exacerbation once a year for 10 years. The 10 possible health states were 100, 110, 200, 210, 201, 211, 300, 310, 301, and 311. The profiles 101 and 111 were not evaluated in this study, because we assumed that serious exacerbation is rare in the moderate COPD according to the results of consultation with COPD specialists. An operational definition of non-serious exacerbation and serious exacerbation was described on the questionnaire.

### Interviews

First of all, respondents evaluated 10 COPD health states on a 20-cm vertical VAS in which the endpoints were labeled ‘100 = Best imaginable health state’ and ‘0 = Worst imaginable health state’. Death was finally evaluated after evaluation of 10 hypothetical health states. Next, they evaluated 10 COPD health states using the TTO method after dividing those states into “better than death” states and “worse than death” states. For “better than death” states, the interviewers attempted to determine a respondent’s point of indifference between the length of time (t) in the best imaginable health state and 10 years in the target health state. “Worse than death” states were not evaluated to reduce the cognitive burden of respondents in the TTO method. In both valuation methods, the respondents were asked to imagine living in each target health state for 10 years. The smallest tradable unit in the TTO method was one year and a ping-pong approach was applied [16]. Furthermore, we used the duration in full health for the response scale and visual aids were not used.

## Analysis

### Calculation of utility weights

Utility weights on VAS for the health states were given by the formula *(x-d)/(100-d),* where *x* was the scale placement of the health state and *d* was the scale placement of death [17]. With the TTO method, utility weights for states ‘better than death’ were calculated using the formula *t/10*, where *t* represented the number of years spent in the best imaginable health. Utility weights for states ‘worse than death’ were given a zero. Possible TTO utility weights ranged from 0 to 0.992. Logical consistency was applied for each given pair of health states, i.e. if one state of a pair is clearly better than the other state, after which the evaluation for the former state must be at least as good as the valuation for the later state [18]. For example, a health state of ‘200’must be at least as good as a health state of ‘301’.

### Statistical analysis

To assess the comparability of the two measurement techniques at the aggregate level, we assessed the mean difference in score between the two techniques [19]. The Wilcoxon rank sum test was also used to evaluate the null hypothesis of an average mean difference of zero, and the Spearman correlation coefficient was used to evaluate the relationship between the differences in the utility scores and the mean utility scores. Because the data were a sample of the population, linear mixed models at the individual level were used to estimate the VAS and TTO utility weights in order to predict values for other members of that population. The dependent variable was the VAS or TTO utility weight. The basic explanatory variables were the severity of the COPD health state and the occurrence of non-serious exacerbation or serious exacerbation, both of which were treated as dummy variables. We also explored models with interaction effects between the COPD severity and the occurrence of exacerbation and/or variables such as patient age, sex, education, and self-perceived health status. The predictive ability of the model was assessed using the mean absolute error (MAE which is the average of the absolute differences between the observed and predicted values and is considered to be an important model selection indicator. A smaller MAE indicates a better model. Generalized R^2^ was also determined in order to assess goodness of fit in the prediction models. Statistical analyses were conducted using the SAS software (ver.9.1, Cary, NC), and P < 0.05 was considered to be statistically significant.

## Results

Among the 323 study subjects contacted for interviews, 200 interviews (61.9%) were successfully conducted. The demographic characteristics and self-perceived health state for survey respondents and the Korean National Health and Nutritional Examination Survey III (KNHANES III) [20] are presented in Table 1. The mean age of study participants was 41.3 years (SD ± 11.3), and 51% were women. The mean EQ-5D index of the study subjects was 0.978 (SD ± 0.05). Problems reporting the rate in each dimension were less than in those from the KNHANES III. A total of 22 physicians (mean age, 41.2 [SD ± 4.8], 27% women] participated in the interviews.

**Table 1:** **Demographic characteristics of the survey participants and national data**

**Characteristics**	**All study respondents**	**KNHANES III** ^**a**^
	**N (%)**	**%**
Sex		
Female	102 (51.0)	50.7
Age		
20–29	46 (23.0)	23.4
30–39	48 (24.0)	24.2
40–49	44 (22.0)	23.4
50–59	39 (19.5)	17.9
60 and older	23 (11.5)	11.0
Level of education (years)		
9 and below	13 (6.5)	20.9
10 to 12	95 (47.5)	41.4
13 and more	92 (46.0)	37.8
Reported problem in the EQ-5D dimension		
Mobility	3 (1.5)	8.4
Self-care	1 (0.5)	1.6
Usual activities	4 (2.0)	5.2
Pain/discomfort	28 (14.0)	21.9
Anxiety/depression	22 (11.0)	12.6
EQ-5D index, mean (SD)	0.978 (0.05)	0.943
EQ-VAS, mean (SD)	82.7 (11.8)	73.9

^a^Korean National Health and Nutrition Examination Survey III (2007 ~ 2009) data. The frequencies and means are weighted based on the population distribution.

All data fulfilled logical consistency requirements and were therefore included in the analysis. There were no statistically significant demographic factors and self-perceived health status variables seen as explanatory variables in the model. Therefore, the model using basic explanatory variables in both the VAS and TTO methods was selected as the preferred model. Parameter estimates for models with VAS and TTO values in the general population and for health professionals are presented in Table 2. In the general population, the VAS values ranged from 0.750 in 100 (moderate COPD without exacerbation) to 0.046 in 311 (very severe COPD having one, non-serious exacerbation and one, serious exacerbation per year). The TTO values ranged from 0.824 in 100 to 0.077 in 311. In the TTO model, the decreases in utility were estimated to be 0.082 for a non-serious exacerbation and 0.228 for both a non-serious and serious exacerbation. The MAE was 0.008 (SD, 0.006) in the VAS model and 0.008 (SD, 0.007) in the TTO model. The generalized R squares were 0.83 and 0.86 in the VAS and TTO models, respectively. Among factors, the severity of ‘severe’ had the largest impact in all models. In health professionals, the regression coefficients in the VAS and TTO models were similar to those of the general population.

**Table 2:** **Parameter estimates for the VAS and TTO models using a linear mixed model in the general population and in health professionals**

**Parameter**	**Visual analogue scale**			**Time trade-off**		
	**Coefficient**	**(SE)**	**p-value**	**Coefficient**	**(SE)**	**p-value**
General population						
Intercept = moderate COPD	0.750	0.009	<0.0001	0.824	0.011	<0.0001
Severe COPD	−0.179	0.005	<0.0001	−0.178	0.006	<0.0001
Very severe COPD	−0.482	0.005	<0.0001	−0.519	0.006	<0.0001
One non-serious exacerbation	−0.078	0.004	<0.0001	−0.082	0.005	<0.0001
One serious exacerbation	−0.155	0.005	<0.0001	−0.164	0.006	<0.0001
One non-serious and one serious exacerbation	−0.217	0.005	<0.0001	−0.228	0.006	<0.0001
Generalized R^2^	0.83			0.86		
MAE, mean (SD)	0.008	(0.006)		0.008	(0.007)	
Health professionals						
Intercept = moderate COPD	0.759	0.027	<0.0001	0.883	0.025	<0.0001
Severe COPD	−0.226	0.014	<0.0001	−0.234	0.016	<0.0001
Very Severe COPD	−0.496	0.014	<0.0001	−0.590	0.016	<0.0001
One non-serious exacerbation	−0.072	0.012	<0.0001	−0.070	0.014	<0.0001
One serious exacerbation	−0.147	0.014	<0.0001	−0.148	0.017	<0.0001
One non-serious and one serious exacerbation	−0.196	0.014	<0.0001	−0.207	0.017	<0.0001
Generalized R^2^	0.93			0.88		
MAE, mean (SD)	0.004	(0.003)		0.011	(0.008)	

**Figure 1:**
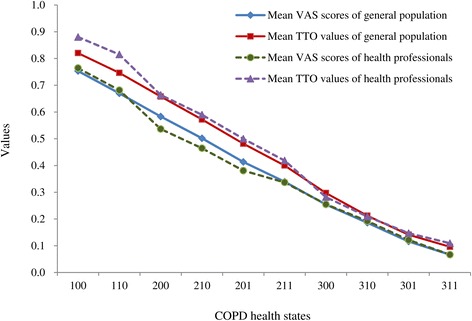
**Observed visual analogue scale (VAS) and time trade-off (TTO) values for COPD health states according to the type of respondent.**

The observed and estimated utility weights for the 10 COPD health states are shown in Table 3. In this study, the COPD state of 111 and 211 showed the largest absolute difference between the observed weights and estimated weights at 0.016 in the VAS and 0.019 in the TTO model. The average difference between the observed mean TTO and the VAS values was 0.054, and which was statistically significant in the general population (p = 0.005) in which the VAS and TTO means were the proportionate differences between the TTO and VAS means (Spearman correlation ρ = 0.84, p = 0.002). The TTO values were higher than the VAS values for all health states in both the general population and in health professionals (Figure 1).

**Table 3:** **Observed and predicted mean values of 10 COPD health states in the general population**

**COPD health states**	**Visual analogue scale**			**Time trade-off**		
	**Observed**	**Predicted**	**Difference**	**Observed**	**Predicted**	**Difference**
Moderate state without exacerbation	0.753	0.750	0.003	0.820	0.824	−0.004
Moderate state with 1 non-serious exacerbation	0.670	0.673	−0.003	0.746	0.743	0.004
Severe state without exacerbation	0.583	0.572	0.011	0.658	0.646	0.012
Severe state with 1 non-serious exacerbation	0.502	0.494	0.007	0.572	0.565	0.007
Severe state with 1 serious exacerbation	0.413	0.416	−0.003	0.482	0.482	0.000
Severe state with 1 non-serious +1 serious exacerbation	0.339	0.355	−0.016	0.400	0.419	−0.019
Very severe state without exacerbation	0.254	0.268	−0.014	0.297	0.305	−0.008
Very severe state with 1 non-serious exacerbation	0.186	0.191	−0.004	0.212	0.224	−0.011
Very severe state with 1 serious exacerbation	0.116	0.113	0.003	0.141	0.141	0.000
Very severe state with 1 non-serious +1 serious exacerbation	0.067	0.051	0.016	0.096	0.077	0.019

## Discussion

In this study, the TTO and VAS values for the COPD health states were elicited from 200 respondents of the general population in South Korea using random effects regression. The value differences between the valuation techniques in the respondent groups were also investigated. A model using the VAS scores as dependent variables showed a good predictive ability and goodness of fit comparable to those of the TTO model. However, substantial differences were noted between the TTO values and the VAS scores. Furthermore, VAS scores are prone to bias and do not use an interval scale of preference [21,22]. For these reasons, a model using TTO values was recommended to calculate the utility weights.

In the TTO model, all regression coefficients were statistically significant and showed logically consistent predictions, i.e. TTO values decreased with increasing severity of the COPD health state and the extent of exacerbation. Very severe COPD showed the largest influence on utility estimates. The difference in the utility estimates between severe and very severe COPD was approximately three times larger than the difference between moderate and severe COPD. Our study showed lower utilities in moderate (0.824), severe (0.646), and very severe COPD (0.305) compared to the values (0.929, 0.717, and 0.522, respectively) previously reported in a study by Rutten-van Mölken et al. and which was based on a profile approach [7].

Health professionals also deemed the extent of the utility decrease with the increasing severity of COPD profiles and frequency and the severity of exacerbation to be larger than did the general population. However, the difference was not statistically significant. The utilities elicited from health professionals were used to assess the preference of doctors for recommending certain therapeutic options to patients. We hypothesized that because health professionals personally witness the patient’s course of COPD, they gave poorer scores than did the general population group which simply imagined hypothetical disease scenarios. Similarly, Owens et al. reported that in the case of HIV, physicians gave lower quality of life scores for severe conditions than did patients [23], as reported in a study by Tsevat et al. [24].

In this study we compared COPD utilities according to the GOLD stage which was measured by EQ-5D applying a UK-based algorithm. Rutten-van Mölken et al. [25] and Stahl et al. [26] reported mean utility scores of 0.787, 0.750, and 0.647 and 0.73, 0.74, and 0.52, respectively, for moderate, severe, and very severe COPD. A systematic review reported pooled mean utility scores according to the GOLD stage of 0.74 (range, 0.66–0.83) in moderate, 0.69 (0.60–0.78) in severe, and 0.61 (0.44–0.77) in very severe COPD [27]. The value for moderate COPD in both studies was lower than that of our present study, while the value for severe and very severe COPD in both studies was higher than that of our present study. A possible reason for these discrepancies is that the classification of the GOLD stage was based on the pulmonary function test in previous studies, while our current classification was based on various dimensions, including breathlessness and the impact of daily activities. In addition, a previous study reported only a weak correlation between the forced expiratory volume per second and the patient’s HRQOL [28].

Exacerbation contributes considerably to the total costs of COPD [29,30], and utility weights at exacerbation are critical to the CUA analysis of COPD. However, reported utility weights at exacerbation have been highly variable in previous economic evaluation studies of COPD. In his model, Borg et al. applied utility decrements of 5% to 70% (0.045 to 0.384) of the original QALY (quality-adjusted life year) weights depending on the severity of exacerbation based on an expert opinion [31]. Oostenbrink et al. applied a utility value decrease of 15% in non-severe exacerbation and 50% in severe exacerbation (0.113 to 0.274) [8]. Sin et al. assumed the reduction to be 0.32 QALYs for each exacerbation episode regardless of the severity of the exacerbation [9]. In a study by Rutten-van Mölken et al. using a profile-based approach, the estimated annual utility decrements were 0.010 and 0.042 for one non-serious and one serious exacerbation per year, respectively, while it was 0.088 for having both one serious and one non-serious exacerbation per year [7]. The utility decrements of exacerbation in our study appear to be much larger, ranging from 0.082 for one non-serious exacerbation to 0.228 for having both one serious and one non-serious exacerbation per year. The reason for these differences in utility weights is unclear and may be partly attributed to cultural divergence and differences in the survey methodology, i.e. questionnaire format and application method. In our study, there was no interaction between the severity of COPD and the extent of exacerbation, such that utility decreases due to exacerbation were similar regardless of the COPD severity. This finding has been observed in previous studies [7,32].

The age and gender of the respondents in this study were similar to those of the Korean general population, while the educational level in study respondents was inclined to be higher than those of the general population. The effects of the educational level on evaluation tasks remain unknown. Dolan et al. reported that the TTO evaluation process was insignificantly affected by education [33], whereas one Spanish study found that the level of education did influence the values of health states [34]. In our model, as the educational level was not found to be a statistically significant variable, the impact of the differences in educational level may not be critical. Finally, we did not collect morbidity information of respondents as a limitation. In this reason, we cannot exclude the opportunity of including COPD patients in the survey, but we expected that such possibility might be very low.

In conclusion, the utilities of various COPD health states are likely dependent on the severity and exacerbation of COPD. No differences were found between our respondent groups, although systematic differences existed between the VAS and TTO scores. Therefore, intervention for the delay of disease progression and appropriate prevention or management of exacerbation in COPD will be helpful in order to improve the HRQOL in COPD patients.

## Additional file

Additional file 1:
**Appendix 1.** Descriptions of the COPD patients’ health status according to the severity. **Appendix 2.** Descriptions of exacerbation according to the severity.
