# Supplementary material for: Utility estimation of hypothetical chronic obstructive pulmonary disease health states by the general population and health professionals
Source: Health Qual Life Outcomes. 2015 Mar 13;13:34. doi: 10.1186/s12955-015-0228-2 (PMC4381507; doi:10.1186/s12955-015-0228-2)
Supplement: Additional file 1: — Appendix 1. Descriptions of the COPD patients’ health status according to the severity. Appendix 2. Descriptions of exacerbation according to the severity. [file 12955_2015_228_MOESM1_ESM.docx]

Appendix 1.Descriptions of the COPD patients’ health status according to the severity

|  | Moderate COPD | Severe COPD | Very severe COPD |
| --- | --- | --- | --- |
| Frequency of dyspnea | Patients are sometimes short of breath. | Patients are often short of breath. | Patients are always short of breath, even at rest. |
| Impact on daily activities | Daily activities are very slightly limited | Patients have to stop for breath after walking for a few minutes | Patients are breathless when dressing or washing |
| Impact on strenuous  Activities | Strenuous activities, such as climbing stairs and playing sports, e.g. football, can cause breathlessness. | Strenuous activities always cause breathlessness. Playing sports such as football is no longer possible. | Climbing stairs as well as playing sports is no longer possible |
| Ability to work | Most patients are still capable of working. Patients whose work  involves heavy physical activity have to give up this type of work. | Many patients have to give up work. | All patients have to give up work. |
| Anxiety and  Depression | Patients are not anxious or depressed. | Patients are often anxious or depressed. | Patients are always anxious or depressed. |
| Energy and  Tiredness | Patients sometimes feel tired. | Patients have less energy and are often exhausted. | Patients have very little energy and quickly become exhausted. |

Appendix 2. Descriptions of exacerbation according to the severity

|  | Non-serious exacerbation | Serious exacerbation |
| --- | --- | --- |
| Symptoms | - Patients experience mild to moderate worsening of their symptoms, especially breathlessness and cough. - Some patients suffer from increased fatigue and insomnia. - The symptoms interfere with daily activities. | - Patients experience severe to very severe worsening of their symptoms, especially breathlessness and cough. - Many patients have fever and suffer from severe fatigue and insomnia. Anxiety and impaired consciousness may also exist. - Daily activities are completely disrupted by the symptoms. |
| Treatment | - Patients should increase their existing respiratory medication or speak to their physician. | - The symptoms worsen to the point where hospitalization is required. Oxygen therapy and ventilation support may be necessary during hospitalization. |
| Duration | - The symptoms last for approximately 10 days. | - The symptoms last for approximately one month. |
